# Electrosurgery reduces blood loss and immediate postoperative inflammation compared to cold instruments for midline celiotomy in dogs: A randomized controlled trial

**DOI:** 10.1111/vsu.12641

**Published:** 2017-03-17

**Authors:** Lee B. Meakin, Jo C. Murrell, Ivan C. P. Doran, Toby G. Knowles, Michael S. Tivers, Guillaume P. A. Chanoit

**Affiliations:** ^1^ School of Veterinary Sciences University of Bristol Langford House Langford Bristol BS40 5DU United Kingdom

## Abstract

**Objectives:**

To compare the use of an electrosurgical device with traditional cold instruments (scalpel and scissors) for midline celiotomy incision.

**Study design:**

Prospective randomized controlled clinical trial.

**Sample population**: One hundred and twenty client‐owned dogs undergoing abdominal surgery.

**Methods:**

Dogs were prospectively recruited and randomized to receive electroincision or cold instrument incision. For cold incision, surgeons used basic surgical instruments including scalpel and scissors. For electroincision, surgeons only used the electrosurgical device in cutting mode. Time for the approach, blood loss, and the incision length were recorded. A blinded observer assessed pain and incision redness, swelling, and discharge at 24 and 48 hours postoperative (graded 0‐3). Owner assessment of incision healing was recorded by telephone interview.

**Results:**

Blood loss during surgery was significantly lower for electroincision (mean 0.7, SD 1.7 mL) than cold incision (mean 3.0, SD 4.3 mL, *P* < .0001) with no significant difference in incision length or time for approach. Electroincision was associated with significantly less incision redness (cold median 1, range 0‐3; electroincision median 0, range 0‐2, *P* = .02) and less incision discharge (cold median 0.5 range 0‐3; electroincision median 0, range 0‐1, *P* = .006) at 24 hours postoperative. There was no significant difference in pain scores or incision healing in dogs receiving the two techniques. No incisional hernias were reported. A surgical site infection occurred in 1 dog (cold incision).

**Conclusions:**

Electroincision for a celiotomy approach in the dog reduces blood loss, and incision redness and discharge in the immediate postoperative period without affecting the occurrence of wound complications such as infection and dehiscence (including linea alba).

## 1 Introduction

Electrosurgical devices were originally designed by William T. Bovie and first used in a person in 1926.^1^ Modern electrosurgical devices function by applying radiofrequency energy through alternating electrical currents. The generated current passes either between 2 electrodes built into forceps (bipolar) or from a single electrode built into a hand piece and through the patient to an earth plate (monopolar). Monopolar electrosurgery handpieces have several settings including cut and coagulation mode.^2^ Cut mode provides a continuous lower voltage current enabling tissue transection through cellular vaporization, while minimizing desiccation, fulguration (tissue ablation), deep thermal injury and charring. The coagulation mode delivers an interrupted, dampened, and relatively high‐voltage waveform which causes more collateral tissue damage. Blend modes are intermediate modes between the cut and coagulation and are also available.

A midline celiotomy approach for abdominal surgery is standard in veterinary surgery. Hemostasis of small perforating subcutaneous vessels can be performed with electrocoagulation but cold instruments (ie, scalpel blade and scissors), are recommended by some for critical steps such as the skin and linea alba incisions.^3^ This recommendation is linked to concerns that electrosurgical devices interfere with healing, especially the linea alba, and may increase occurrence of wound infection or dehiscence.^4^ A study in rats showed that electroincision of the abdomen resulted in significantly lower tensile strength of the incision compared to a scalpel alone.^5^

Nevertheless, electrosurgical devices are commonly used to perform all aspects of the abdominal approach in people, including the skin incision^6^ and abdominal wall incision.^7^ There is strong evidence that electrosurgery does not increase incision infection, incisional hernia, or worsen cosmesis, but does reduce blood loss, incision time, and early postoperative pain.^4^, ^6^, ^8^, ^9^

The evidence in animals mostly relates to experimental rodent studies, which demonstrate reduced incision healing compared with scalpel incisions^5^, ^10^, ^11^ or no difference in healing.^12^ A recent study in rabbits showed that monopolar electrosurgical devices used in coagulation mode induced poorer tissue healing than when used in cut mode.^11^ Differences in skin and body wall physiology and anatomy restrict extrapolation of these results to companion animals.^13^ In companion animals, the use of scalpel and electrosurgical devices has been compared for soft palate resection^14^ and for histologic quality of skin biopsies.^15^ We aimed to compare electrosurgery to cold instruments for the midline celiotomy approach in dogs, comparing aspects of the surgical approach, postoperative pain, incision inflammation, and healing. We hypothesized that electroincision would provide superior hemostasis and reduce incision time with no detrimental effect on postoperative pain, incision inflammation, or healing.

## 2 Materials and methods

The study was reviewed and approved by our Institutional Ethical Review Committee (VIN/13/047). One hundred and twenty dogs undergoing a standard midline celiotomy for a variety of abdominal procedures were prospectively enrolled. This number was based on a sample size calculation to detect a 15% difference in incision complication occurrence with 90% power based on a 5% occurrence of incision complications including infections.^16^ The signalment, reason for surgery, and National Research Council (NRC) wound classification^17^ were recorded. Dogs were randomized by permuted block randomization in blocks of 4 to receive either cold incision or electroincision. The primary surgeon was one of 4 board certified surgeons or a resident under their supervision. For cold incision, surgeons had access to basic surgical instruments including scalpel and scissors but electrosurgery was not available until the approach had been completed, that is, when all tissue layers (skin, subcutaneous tissue, and linea alba) had been incised and the abdominal cavity was fully opened. Once the approach was completed, point application of electrocoagulation for hemostasis was permitted with cold incision. Excision of the falciform ligament was not considered part of the approach in this study but was performed in some dogs once the abdominal approach had been completed. Suture material was available for ligation of larger vessels and bleeding from smaller vessels was controlled using pressure or artery forceps. For electroincision, surgeons exclusively used the electrosurgical device (monopolar electrode) in cut mode to incise all tissue layers (skin, subcutaneous tissue, linea alba) without access to a scalpel blade or scissors. The time taken for completion of the approach, blood loss during the approach, and the incision length were recorded. Blood loss was estimated by weighing used swabs and dry swabs at the end of the approach using the conversion of 1 g = 0.94 mL of blood.^18^

The electrosurgical unit was a Force 2 unit (Valleylab, Medtronic, Minneapolis, Minnesota) or an Eschmann 10 series (West Sussex, United Kingdom). The handpieces were all disposable monopolar pencils with an inbuilt cut and coagulation button and a spatula tip (finger switch pencil, Eschmann). Closure of the celiotomy was with polydioxanone for the linea alba in a simple continuous pattern and poliglecaprone for the subcutaneous layer in a simple continuous pattern. The skin was apposed using poliglecaprone in the dermis in a simple continuous pattern or monofilament nylon in the skin in a Ford interlocking pattern. Suture material size was at the discretion of the primary surgeon.

The anesthesia protocol was determined by the primary anesthetist but no local analgesia techniques were permitted, obviating epidural, local nerve blocks, splash blocks, and wound soaker catheters. All dogs received an opioid (fentanyl, methadone, or buprenorphine) and a non‐steroidal anti‐inflammatory drug (meloxicam or carprofen) or paracetamol postoperative, when appropriate.

A blinded observer assessed dogs at 24 and 48 hours postoperative. Pain was recorded based on the Glasgow Composite Pain Score (24‐point scale)^19^ and incisions were subjectively scored for redness, swelling, and discharge using a 4‐point scale (0 = none, 1 = mild, 2 = moderate, 3 = severe).^20^

Owners were telephoned in the postoperative period to question whether the incision had fully, partially (<50% dehiscence), or not healed (>50% dehiscence) at the time of anticipated suture removal (10 days postoperative), or whether the incision was red, swollen, or discharging at this time based on the scale described above. Any instances of suspected postoperative infections or other complications such as incisional hernia were also recorded. Referring veterinary practices were telephoned if owners were not contactable.

### 2.1 Statistical analysis

Continuous responses were assessed for normality using the Kolmogorov‐Smirnov test. Continuous data were compared between cold and electroincision using unpaired *t* test. Ordinal or categorical data were compared between cold and electroincision using the Mann‐Whitney U or chi‐square tests, respectively. Significance was set as *P* < .05. Continuous data are reported as mean (SD) with 95% CI for the difference in means. Ordinal and categorical data are reported as median (range). Statistical analysis was performed using SPSS for Windows (version 23; SPSS Inc, Chicago, Illinois).

## 3 Results

The study enrolled 120 dogs with a mean (SD) bodyweight of 19.5 (12) kg and mean (SD) age of 6.1 (4) years. There were 63 female dogs (18 entire, 45 neutered), 55 male dogs (21 entire, 34 neutered), and 2 hermaphrodites (1 entire, 1 neutered). There were 41 different breeds (13 Labrador Retriever, 13 Cocker Spaniel, 7 Springer Spaniel, 5 Border Collie, and <5 for each other breed). There was no difference in the signalment of dogs receiving cold or electroincision (bodyweight *P* = .21, breed *P* = .63, sex *P* = .56, age *P* = .54). Abdominal surgery was for conditions of the gastrointestinal tract (n = 38), hepatobiliary system (29), urogenital system (37), splenectomy (10), and other (6). There was no significant difference in the reason for surgery between dogs receiving cold or electroincision (*P* = .60). The NRC wound classification was clean (n = 43), clean‐contaminated (68), contaminated (3), and dirty (6). There was no significant difference in the frequency of NRC wound classification between dogs receiving cold or electroincision (*P* = .70).

The time taken to complete the approach was not significantly different between dogs receiving cold or electroincision; cold 3.9 (3.1) minutes, electroincision 3.3 (1.6) minutes, 95% CI −3.6‐1.4, *P* = .23. The incision length was not significantly different; cold 15.7 (5.2) cm, electroincision 15.6 (4.2) cm, 95% CI −1.6‐1.8, *P* = .91. There was significantly less blood lost for electroincision than cold incision; cold 3.0 (4.3) mL, electroincision 0.7 (1.7) mL, 95% CI 1.1‐3.4, *P* < .0001.

The pain score or swelling 24 hours postoperative was not significantly different between dogs receiving cold or electroincision; pain score cold 1 (0‐8), electroincision 1.5 (0‐7), *P* = .55; swelling cold 0 (0‐3), electroincision 0 (0‐1), *P* = .12. The incision redness and discharge were significantly less for electroincision; redness cold 1 (0‐3), electroincision 0 (0‐2), *P* = .02; discharge cold 0.5 (0‐3), electroincision 0 (0‐1), *P* = .006. The pain score, swelling, incision redness, and discharge were not different at 48 hours postoperative; pain score cold 1 (0‐4), electroincision 1 (0‐5), *P* = .48, swelling cold 0 (0‐2), electroincision 0 (0‐2), *P* = .82; redness cold 1 (0‐2), electroincision 0 (0‐2), *P* = .18; discharge cold 0 (0‐2), electroincision 0 (0‐2), *P* = .26.

Eleven dogs died or were euthanatized prior to suture removal. Telephone follow‐up was possible for 79/109 remaining dogs (72%). All skin incisions progressed to full healing. At 10 days postoperative for planned suture removal, 3 incisions were reported as partially healed (1 cold, 2 electroincision). There was no significant difference in incision redness (*P* = .77), swelling (*P* = .33), or discharge (*P* = .78) at 10 days postoperative between dogs receiving cold or electroincision.

One dog receiving cold incision was reported with incision infection when the dog chewed and opened the incision, which was subsequently stapled. This incision became infected and was managed successfully as an open wound. No other incision infections were reported. There were no reports of any incisional hernia.

## 4 Discussion

We showed the use of electroincision for the skin, subcutaneous tissue, and linea alba was comparable to cold instruments for most measured parameters but was beneficial with less blood loss. This benefit has been previously reported in people.^8^ Although the difference in the volume of blood loss (mean 2.3 mL) is likely clinically irrelevant, it may be important in animals with anemia or coagulopathy. Furthermore, bleeding during the approach can impair visualization. Surgeons were allowed to coagulate bleeding vessels following completion of the approach for either technique. When electrosurgical devices are not available, ongoing and possibly unnoticed hemorrhage could contribute to further blood loss. Hemorrhage can potentially go unnoticed due to the presence of abdominal swabs and abdominal retractors placed for exposure during abdominal procedures.

There was no significant difference in the time taken for the celiotomy in our study despite reports in people citing that electrosurgery significantly reduces the incision time.^6^, ^8^ Our study included multiple surgeons, many who do not routinely use electrosurgery for the entire abdominal approach, which may have incited a learning curve. Although we did not include excision of the falciform ligament as part of the study protocol, the authors find electrosurgical excision of the falciform ligament is advantageous. Falciform ligament excision was not included since it was not removed in all animals, and inconsistency in the size and vascularity may have affected results.

There was no significant difference in postoperative pain with all dogs despite receiving similar analgesia. Short‐term postoperative pain is reported to be reduced in people with electrosurgery compared to scalpel based on subjective pain assessment^6^ and the requirement for morphine via patient‐controlled analgesia pumps.^8^ Accurate assessment of postoperative pain in animals is challenging, even with the use of validated pain scores such as the Glasgow Composite Pain Score.^19^ We did not use objective measures of pain assessment such as mechanical nociceptive threshold,^21^ which may have given a different result.

Incision inflammation was subjectively scored based on redness, swelling, and discharge. Only redness and discharge was significantly reduced for electroincision at 24 hours postoperative but was no longer apparent at 48 hours postoperative. The reduced redness and discharge may relate to small vessels being sealed with electrosurgery, therefore reducing postoperative bleeding into the incision (which could be viewed as redness) and also discharge. Postoperative inflammation after electroincision or scalpel incision has not been compared in clinical trials in people or animals. Interestingly, larger zones of inflammation have been observed in animal models after electrosurgery compared to scalpel but only with the coagulation mode rather than cut mode.^10^, ^11^ We used the cut mode exclusively for the incisions. Many surgeons routinely incise tissue using the coagulation mode due to a perceived superior hemostasis, but there is the disadvantage of more collateral tissue damage. The restriction to cut mode standardized the approach and was considered more appropriate for incision, with less potential detrimental effect on wound healing.^10^, ^11^

Surgeons in practice may use a combination of cold surgery with electrosurgery for both cutting and coagulation which will improve hemostasis and reduce blood loss.

All incisions that were followed up eventually went on to heal and there were no reports of incisional hernias. Experimental studies report significantly lower fascial wound strength at 1, 2, and 3 weeks postoperative but no difference was apparent at 6 weeks postoperative, at which time all incisions were as strong as the pre‐incision tissue.^10^ This difference was more pronounced when the incision was made in coagulation mode rather than cut mode. We had 1 dog (cold incision) with postoperative incision infection, considered the result of self‐mutilation. With no complications in any other dog, definitive conclusions could not be made.

The study was limited by subjective postoperative incision examination and pain scoring. However, independent observers did perform these assessments. Final incision assessments were reliant on a telephone description by the owner or referring veterinarian or nurse rather than observation by the same independent observers. Small hernias or subtle differences in incision healing may have been missed but clinically significant complications would have been recorded. We could not assess the cosmetic appearance of the incision beyond the owners' assessment. However, in people, there is no reported difference.^6^

The use of electroincision in cut mode was not associated with any adverse events after midline celiotomy with incisions through the skin, subcutaneous tissue, and linea alba. There were advantages of reduced blood loss during the approach and short‐term reductions in incision redness and discharge with electroincision. The use of electrosurgery should be considered when performing midline celiotomy in dogs.
